# Open Photonics: An integrated approach for building a 3D-printed motorized rotation stage system

**DOI:** 10.1016/j.ohx.2024.e00577

**Published:** 2024-08-28

**Authors:** Yannic Toschke, Jan Klenen, Mirco Imlau

**Affiliations:** Department of Physics, Osnabrueck University, 49076 Osnabrueck, Germany

**Keywords:** Rotation stage, Optomechanics, Optical components, Photonics, Optics, 3D-printing

## Abstract

In the context of experimental optics- and photonics-research, motorized, high-precision rotation stages are an integral part of almost every laboratory setup. Nevertheless, their availability in the laboratory is limited due to the relatively high acquisition costs in the range of several 1000€ and is often supplemented by manual rotation stages. If only a single sample is to be analyzed repeatedly at two different angles or the polarization of a laser source is to be rotated, this approach is understandable. Yet, in the context of automation and the associated gain in measurement time, cost-effective and precise rotation stages designed for the use of optics are lacking.

We present a low-cost alternative of a motorized high precision rotation stage system. The design is based on a combination of 3D-printed components, which form the monolithic mechanical framework, and a stepper motor controlled by an ESP32 based microcontroller. By coupling the motor and rotation unit via a toothed belt, backlash is minimized and at the same time high positioning accuracy can be achieved. Finally, the implementation of remote procedure calls for serial communication and the utilization of a physical home switch and incremental encoder complete the desired feature set of an integrated system for laboratory setups. The total costs can thus be reduced to less than 100€ without significantly restricting the performance criteria.

## Graphical abstract

**Figure:**
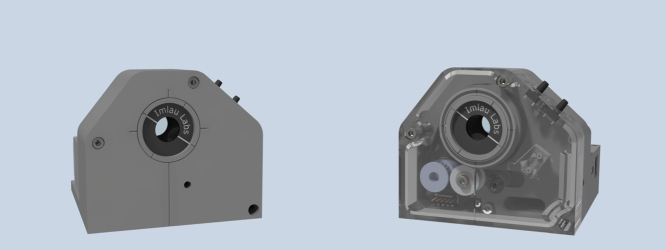

**Table:** 

Specifications table	

**Hardware name**	*Modernized DIY Stepper Motor Rotation Stage*

**Subject area**	•Optics and Photonics
	•Experimental physics

**Hardware type**	•Optomechanical devices

**Closest commercial analog**	K10CR1 – Motorized Rotation Mount for Ø1” Optics, Stepper Motor (Thorlabs, Inc)

**open-source license**	This work is licensed under the Creative Commons Attribution-ShareAlike 4.0 International License.

**Cost of hardware**	< 100€

**Source file repository**	https://osf.io/6gd27/

## 1 Hardware in context

Motorized rotation stages represent an integral part in experimental optics- and photonics-research by precisely rotating optics, detectors and samples alike. Their application ranges from simple devices for intensity regulation to beam steering [1], [2], optical characterization techniques such as ellipsometry or polarimetry [3], [4], [5] over to a variety of other applications in spectroscopy, microscopy or holography [6], [7], [8], to list just a few. These applications impose high demands on the mechanical precision and repeatability. Commercially available rotation stages therefore require high-precision mechanical components, posing a considerable challenge in terms of manufacturing costs. As a result, readily available products prices range from 1000€ up to 5000€ [9], [10], [11]. For this reason, the acquisition of multiple motorized high-precision rotation stages represents a considerable financial hurdle, which is met with manual rotation stages wherever possible. If, for example, a sample only needs to be macroscopically rotated and/or realigned between two measurements, or the polarization of a laser source needs to be rotated using a wave retarder plate, the use of manual rotation stages is a viable option. Nevertheless, motorized rotation stages offer a clear advantage in the form of automation, resulting in a time conserving utilization of elaborate and expensive laboratory systems. However, the superficial solution to the automation problem of utilizing more cost-effective commercial motorized rotation stages fails because of their lack of availability. DIY approaches, which are specifically geared towards the field of photonics, therefore offer a noteworthy alternative.

In recent years and with the increased availability of 3D-printers, there has been a push towards open-source hardware by hobbyists and researchers [12]. In addition to gadgets, tools and art, platforms such as UltiMakers’s thingiverse.com or the Flamingo Project, already offer devices for laboratory use [13], [14]. In addition, scientists in the field of photonics participate in this development, for example by publishing peer-reviewed toolkits to realize optical setups like the UC2 project, μCube or Optocubes [15], [16], [17], [18].

Already existing open-source approaches of motorized rotation stages often rely on a mixture of 3D-printed components in combination with precision stepper motors and controllers originally developed for 3D-printers [19], [20], [21]. The devices are highly adaptable and can be used as both a rotation and rotary stage, for example, while offering precision movement with accuracy in many instances comparable to its commercial counterpart. All this is available at a significant reduction of cost, with prices ranging from 200€ to 300€ [22].

Despite these efforts, the current open-source alternatives still come along with some significant drawbacks. Although the hardware components of the systems have a relatively mature status, the combination of hardware and software is often immature. Therefore, the open-source hardware is far from a robust, monolithic system that can be used practically and flexible. In particular, the integration of electronic components is only rudimentary, as it remains at the level of a prototype. Additionally, crucial features like a hardware sided homing feature or an encoder to monitor step loss and provide information about the actual position, are missing. At the same time, these devices lack a modern approach on the software and hardware sided integration.

In this context, a low cost, open-source rotation stage for use in photonics is presented. The design consists of 3D-printed components and few mechanical parts. A small sized stepper motor, controlled by a miniaturized implementation of the ESP32 microcontroller and a compact stepper motor controller, allows for an overall compact design of the hardware. A key advantage is the integration of the electric control unit within the rotation stage, which is enabled by a custom tailored PCB design. These advantages are combined with a modernized communication approach that relies on synchronized serial communication of the control unit with the ESP32 via the remote-procedure-call (RPC) and a USB-C connector [23], [24]. Despite this, the overall system is highly cost-efficient, with a total cost of below 100€.

## 2 Hardware description

**Fig. 1:**
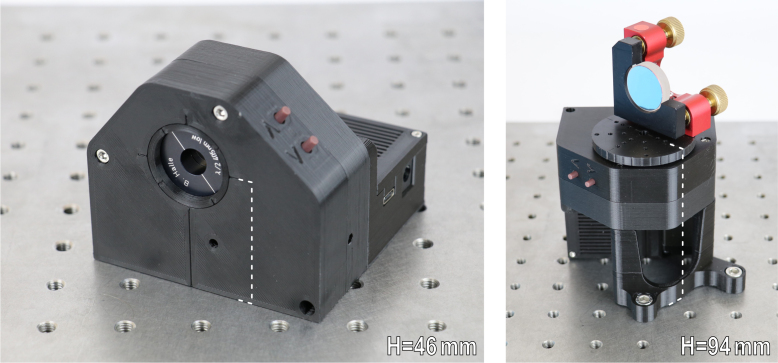
Shown are the photographs of the fully assembled rotation stage in both of its possible configurations. Left: Configuration for 1 inch optics with transmission along the axis of rotation at a height of 46 mm, here a 405 nm half wave retarder plate. Right: Configuration for optics, filter glasses or samples perpendicular to the axis of rotation at a height of 94 mm, here a mounted silver mirror.

The herein presented motorized rotation stage is designed as an easy-to-use system that can be employed as a standalone system or linked with others. The RPC communication allows synchronized, sequential control and avoids interference of different function calls. The hardware is easy to manufacture and assemble. It relies on commonly available mechanical components and additively manufactured parts. For the motorization, different hardware components are used. These include an ESP32 based microcontroller (Seeed Studio XIAO ESP32C3 Tiny MCU Board), a compact stepper motor (NEMA11) with a dedicated stepper controller (Bigtreetech TMC2209), a mechanical home switch (Omron D2AW), an integrated encoder (CUI AMT10E3-V) and pushbuttons. Power is provided by a 12 V DC power supply. The corresponding socket is integrated in the housing and accessible from the outside. A custom designed printed circuit board (PCB) allows for a compact and integrated design of the electronics within the rotation stages casing. The use of wire to board connectors eases the (dis)assembly process. The assembled rotation stage in both of its possible configurations is shown in Fig. 1.

Unlike other open source implementations, our approach features several key improvements. These include a highly cost-effective design that undercuts existing alternatives by more than a factor of two. Despite the reduced cost, the system is on par with its counterparts with regard to precision and accuracy. A modernized approach to software-based integration based on a remote procedure call (RPC) and an easy-to-use Python implementation is combined with the common and widely available USB-C standard for communication. The RPC allows for synchronized communication and effortless integration into preexisting measurement infrastructures. A key feature is the fully integrated and compact design. The monolithic system, with all components integrated into one device, is comparable to commercial devices, but has not been seen in open source implementations until now. In particular, the use of an integrated circuit board allows bypassing the cable management challenges present in other devices. This results in increased ease of use and integration into a measurement setup. In addition, the device features a physical home switch, which eliminates the need for an inconvenient calibration of the device before each measurement, as it is the case when using a software-based home switch. Finally, the inclusion of an incremental encoder, provides a crucial advantage in high microstepping/resolution scenarios by identifying step-losses and potential outliers. In conclusion, these features combined improve on other existing open source implementations by providing not only convenient but necessary additions.

• The system is highly cost-efficient at ≈100€, while offering a comparable 1.0 mrad precision and 156 μrad resolution like commercial and open-source alternatives.
• The systems offers the crucial implementation of a physical home-switch, external control buttons and an incremental encoder.
• The communication is based on a modernized approach, utilizing remote procedure calls (RPC) for an effortless integration into different software architectures.
• The system has a small footprint and features a fully integrated controller with built in USB-C data and 3.5 mm power connection on a single PCB.
• The system offers multiple mounting options, either directly onto an optical table or on an optical post.
• The systems rotational axis can be easily utilized inline as well as perpendicular to any incident laser beam by converting it to a platform configuration.

## 3 Design files summary

This section features a listing of all files for 3D-printing, the files associated with the printed circuit board manufacturing and the microcontroller software (see Table 1). Each 3D-printed component is provided with a consecutive two digit numerical designator XX. A technical drawing of the rotation stage referencing these designators can be found in Fig. 3 of Section 5 ’Build instructions’. As the number of 3D-printed components utilized is relatively small, please refer to the self-explanatory filenames for a more detailed description of the corresponding part. The design file structure can be roughly divided into the casing, the conversion kit and miscellaneous parts. Of those, the only critical component is the 63T_gear (05), whose outer diameter should be as close to 39.6 mm as possible. Additionally, for 3D printing, a 0.25 mm nozzle combined with 100% infill was utilized to achieve sufficient detail for the teeth. In addition, the design files contain all the files for the prefabricated printed circuit board that controls the rotation stage. Production can be carried out by service providers such as JLCPCB, who manufacture the partially assembled PCB.

**Table 1:** Rotation stage design files.

Designator	Design filename	File type	open-source license	File location
01	‘RStage_Casing_Front’	.stl	CC BY-SA 4.0 Deed	https://osf.io/
02	‘RStage_Casing_Back’	.stl	CC BY-SA 4.0 Deed	https://osf.io/
03	‘RStage_Casing_ESP32C3’	.stl	CC BY-SA 4.0 Deed	https://osf.io/
04	‘RStage_Casing_ESP32C3_Cover’	.stl	CC BY-SA 4.0 Deed	https://osf.io/
05	‘RStage_63T_Gear’	.stl	CC BY-SA 4.0 Deed	https://osf.io/
06	‘RStage_Belt_Tensioner’	.stl	CC BY-SA 4.0 Deed	https://osf.io/
07	‘RStage_Button_Retainer’	.stl	CC BY-SA 4.0 Deed	https://osf.io/
08	‘RStage_Enc_Retainer’	.stl	CC BY-SA 4.0 Deed	https://osf.io/
09	‘RStage_ConvKit_Plate’	.stl	CC BY-SA 4.0 Deed	https://osf.io/
10	‘RStage_ConvKit_Plate_Lock’	.stl	CC BY-SA 4.0 Deed	https://osf.io/
11	‘RStage_ConvKit_Stand’	.stl	CC BY-SA 4.0 Deed	https://osf.io/
12	‘RStage_ConvKit_Stand_Cover’	.stl	CC BY-SA 4.0 Deed	https://osf.io/

–	‘Optic_Tool’	.stl	CC BY-SA 4.0 Deed	https://osf.io/

–	‘PCBLayout’	.zip	CC BY-SA 4.0 Deed	https://osf.io/
–	‘PCBPickAndPlace’	.csv	CC BY-SA 4.0 Deed	https://osf.io/
–	‘PCBBOM’	.csv	CC BY-SA 4.0 Deed	https://osf.io/

–	‘ESP32_MotionCTRL’	.ino	MIT License	https://osf.io/
–	‘ESP32_MotionCTRL_Py’	.py	MIT License	https://osf.io/
–	‘RotationstageGUI’	.py	MIT License	https://osf.io/

## 4 Bill of materials summary

This section contains a list of all components and consumables, such as filament and wires, required to build one rotation stage system (see Table 2). When calculating the unit cost, the bolts in particular are based on commercially available packages, which may already be available depending on the workshop’s resources. It should be noted that, at least in Germany, individual bolts can be purchased cheaply in hardware stores like ’Hornbach Baumarkt AG’ if required. All bolts used feature a standard/coarse pitch.

The additional tools required are: A 3D-printer, a soldering iron and crimping pliers for Japanese Solderless Terminals (JST). It should also be noted that a suitable already available crimping tool, which is not explicitly designed for JST connectors, can be used. If no 3D-printer is available, it is also possible to order the 3D-printed components by a 3D-printing service provider like JLC3DP for an additional cost of 30€.

**Table 2:** Rotation stage bill of materials.

Designator	Component	Quantity	Unit cost	Total cost	Source of materials
–	Green TEC PRO	0.15 kg	44.99€	8.43€	DAS FILAMENT
–	Thread insert M3 × 5.7	6	0.09€	0.54€	ruthex
13	Stepper 11HS18-0674S	1	9.95€	9.95€	stepperonline
14	TMC2209 RBS13648	1	6.00€	6.00€	roboter-bausatz
15	XIAO ESP32C3	1	6.68€	6.68€	eckstein-shop
16	Printed circuit board	1	7.40€	7.40€	JLCPCB
17	Belt GT2 509-003	1	3.70€	3.70€	HC-Maschinentechnik
18	Ball bearing 6706-ZZ	2	4.58€	9.16€	Kugellager-Express
19	Basic switch D2AW	1	1.60€	1.60€	Mouser Electronics
20	Encoder AMT10E3-V	1	22.18€	22.18€	Mouser Electronics
21	Gear wheel GT2 20t	1	2.50€	2.50€	3D-Druckershop
22	Pulley 0602430616099	1	2.50€	2.50€	3D-Druckershop
23	Bolts M2 × 5 DIN 912	6	0.10€	0.60€	Frantos
24	Bolts M2.5 × 5 DIN 912	5	0.15€	0.75€	Frantos
25	Bolts M3 × 10 DIN 912	1	0.04€	0.04€	Frantos
26	Bolts M3 × 14 DIN 912	1	0.04€	0.04€	Frantos
27	Bolts M3 × 22 DIN 912	6	0.07€	0.42€	Frantos
28	TASTER 3301B	2	0.13€	0.26€	reichelt elektronik
–	JST XH CKB	20	0.13€	2.60€	reichelt elektronik
–	JST XH2P BU	3	0.07€	0.21€	reichelt elektronik
–	JST XH4P BU	1	0.10€	0.10€	reichelt elektronik
–	JST XH5P BU	1	0.11€	0.11€	reichelt elektronik
–	Wire 0.14 mm${}^{2}$	2	0.95€	1.90€	reichelt elektronik
–	Power supply 12 V 1 A	1	7.18€	7.18€	reichelt elektronik
–	USB cable VT-5334	1	1.89€	1.89€	reichelt elektronik

				96.74€	

## 5 Build instructions

This section gives a detailed, step-by-step build instruction of the motorized rotation stage system. It includes the wiring instruction for the printed circuit board, the assembly of the casing, and the setup and initialization of the microcontroller. The following explanations assume a basic understanding of using the ESP32 microcontroller and the Arduino IDE. For beginners, basic introductions to the above applications can be found at the following Refs. [25], [26]. A supplemental video showing the rotation stage’s mechanical assembly is available through the following URL: https://osf.io/.

### 5.1 Electrical wiring

**Fig. 2:**
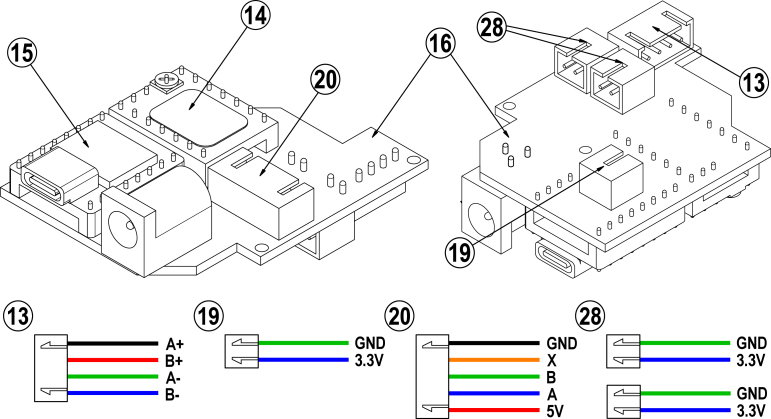
Top: Printed circuited board (16) fully assembled with the stepper controller (14) and the microcontroller (15) soldered in place. Bottom: Wiring guide of the JST XH crimping connectors for the stepper motor (13), the home switch (19), the encoder (20) and the pushbuttons (28).

A central feature of this work is the prefabricated printed circuit board, which controls the rotation stage. The fabrication can be carried out by service providers such as JLCPCB, directly delivering the partially assembled PCB. Given, that the featured PCB is used to wire the different components, only a few steps remain to fully assemble the rotation stage’s control unit. These include the soldering of the stepper motor controller (14, Bigtreetech TMC2209), the ESP32 (15, XIAO ESP32C3), the home switch (19, Omron D2AW) and the pushbuttons (28, TASTER 3301B) as well as crimping the JST XH connectors. In order to provide a comprehensive overview of the electrical wiring, the circuit diagram and the corresponding PCB layout are depicted in Fig. 16 of Appendix B. A condensed and simplified overview is presented in Fig. 2.

1. Solder the contacts of the TMC2209 (14) and the ESP32 (15) to the partially pre-assembled PCB (16) to complete it. The final assembly is shown in Fig. 2 left as a reference.
2. Solder cables of sufficient length to the home switch (19), the pushbuttons (28) and the encoder (20). If necessary, shorten the cables of the stepper motor (13) to 50 mm.
3. For correct crimping, all 15 cables must be stripped 2 mm on the non-soldered side. The cables can then be crimped using the JST XH CKB pins.
4. Finally, the crimped cables must be plugged into their corresponding connectors in the correct position. Fig. 2 on the right shows the pin assignment of all the JST XH connectors.
− Tip: For the stepper motor (13), the pin assignment corresponds to the color code used in the illustration. In the case of the encoder (20), the pin assignment is stamped above the pins.
5. The final assembly now only requires the stepper, encoder, home switch and pushbuttons to be plugged in the PCB and takes place during the assembly of the rotation stage.

### 5.2 Rotation stage assembly

All components listed in the bill of materials, including the fully assembled PCB, are required to build the rotation stage. For the 3D-printed components, it is recommended to utilize a material that provides sufficient heat resistance (e.g. ABS, or PLA with specific additives) to prevent the housing from softening due to the temperatures potentially reached by the stepper motor. Green Tec Pro is used in this work, which has a heat deflection temperature of 115 °C (HDT/B) in addition to its positive printing properties.

1. First take the ESP32 housing (03) and melt the M3x5.7 threaded inserts in the three holes provided for this purpose on the front side of the housing. This can best be done with a soldering iron.
2. Repeat this soldering process for the casing_back (02), the stand (11) and the stand_cover (12) of the conversion kit.
3. Next, take the casing_back (02) and bolt it to the EPS32 housing (03) by screwing two M3 × 22 bolts (27) into the threaded inserts (lower left and upper right).
4. Take the stepper motor (13) and bolt it to the housing_back (02) using the holes provided on the bottom left-hand side. Use four M2.5 × 5 bolts (24) for this purpose. Ensure, that the wires are oriented towards the center of the rotation stage.
5. Slide the green sleeve from the encoder (20) accessories over the shaft of the motor (13) facing away from the stepper and press the corresponding adapter onto the sleeve.
6. Insert the encoder (20) into the casing_back (02) and check that the shaft adapter is flush. If not, repeat the previous step and reposition the sleeve and adapter accordingly.
7. Mount the gear wheel (21) to the motor shaft. A distance of about 1 mm should be maintained between the gear and the encoder (20). Ensure to orient the mounting screws to line up with the flat side of the D-shaft.
8. Insert and bolt down the enc_retainer (08) using two M2 × 5 bolts (23). The encoder (20) should now be fixed into place.
9. Now feed the encoder cables through the hole between the casing_back (02) and the ESP32 housing (03). The cable is plugged in later.
10. Take the wired home switch (19) and press it in the corresponding internal holes of the casing_front (01). Due to the selected tolerances, it retains itself.
11. Next up, take the two wired pushbuttons (28), the corresponding button_retainer (07) and a M2.5 × 5 bolt (24) and mount them into the casing_front (01). The wiring can then be routed in the integrated cable duct.
12. To prepare the 63T_gear (05) for installation, press the two 1 inch (2.54 cm) ball bearings (18) onto the two outer rims of the 63T_gear.
13. Prepare the belt_tensioner (06) by bolting the idler pulley (22) with a M3 × 14 bolt (26) onto it.
14. Place the toothed belt (17) around the 63T_gear (05) teeth and wrap the remaining end around the gear (21) on the motor shaft. Gently press the 63T_gear (05) with the belt combined into the insert of the casing_back (02). There is only one possible orientation of the 63T_gear (05).
15. Now insert the belt_tensioner (06) while applying a minimum of force and loosely bolt it down with the M3 × 10 bolt (25). The belt is tensioned later in step 21.
16. The casing_front (01) is now mounted on the casing_back (02). To do so, the wires of the home switch (19) and the pushbuttons (28) need to be fed through the hole between the casing_back (02) and the EPS32 housing (03).
17. Place the casing_front (01) on top of the casing_back (02) while at the time inserting the 63T_gear (05).
18. If everything lines up, insert the stand_cover (12) in the back of the casing_back (02). Now bolt the casing_front (01) to the casing_back (02) using three M3 × 22 bolts (27).
19. Now take the fully assembled PCB (16) and plug in the stepper (13), the encoder (20), the home switch (19) and the pushbuttons (28). Afterwards, the PCB (16) can be inserted into the ESP32 housing (03) and bolted down using two M2 × 5 bolts (23).
20. To finish the casing, bolt the ESP32 cover (04) to the ESP32 housing (03) utilizing two M2 × 5 bolts (23).
21. Finally, tension the belt through the small hole on the right-hand side of the casing_back (02) and tighten the belt tensioner (06) through the access hole added on the side of the casing_front (01).

**Fig. 3:**
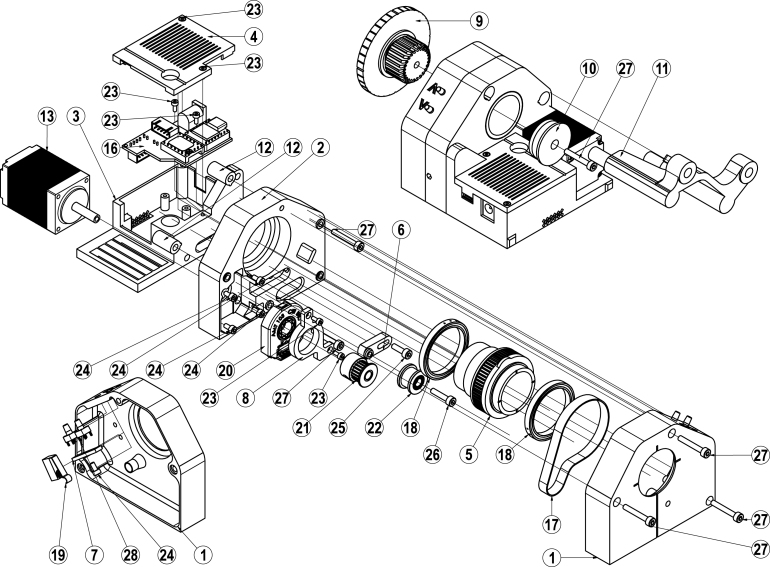
Exploded-view drawing of the motorized rotation stage. The PCB and electronic components are not shown for the sake of clarity.

### 5.3 Rotary stage conversion

The previous section described in detail how to assemble the rotation stage from scratch. This section now describes how to convert the rotation stage to a rotary stage, also known as its platform configuration. For this purpose a simple conversion kit was developed, consisting of only three parts: The conversion kit plate (09), the corresponding plate lock (10) and a stand (11).

1. Unbolt the stand_cover (12) through the two upper screws of the casing_front (01) and gently push them out with the screwdriver.
2. Insert the stand (11) in the back of the casing_back (02) and bolt it down using two M3 × 22 bolts (27).
3. Finally, insert the conversion kit’s plate (09) into the 63T_gear (05) and fixate it with the plate_lock (10) utilizing a M3 × 22 bolt (27).

### 5.4 Flashing the microcontroller

Following the assembly of the rotation stage, the microcontroller software has to be flashed. This section gives an in depth instruction on how to set up the XIAO ESP32C3 microcontroller using the Arduino IDE. An introduction, to set up the serial connection for remote control operation in python is provided in Section 6.2 Section 6.

1. The software for the ESP32 microcontroller is supplied within the ESP32_MotionCTRL file. In order to flash the software to your device, the native Arduino development environment (Arduino IDE) is used.
2. First, the XIAO ESP32 has to be added as a board in the Arduino IDE. For this, navigate to **File** → **Preferences** and add the URL https://raw.githubusercontent.com/espressif/arduino-esp32/gh-pages/package_esp32_index.json to the board manager.
3. Next, the simple RPC library **simpleRPC.h** has to be installed (Tip: simply search for the library in the Arduino IDE).
4. Connect your XIAO ESP32 to your external devices via a USB-C cable.
5. Open the ESP32_MotionCTRL.ino file and navigate to **Tools** → **esp32** → XIAO_ESP32C3, to choose the correct board driver.
6. Ensure that the correct port of the device is selected under **Tools** → **Port**.
7. Finally, compile and upload the software to the microcontroller. Make sure to press the boot button on the ESP32 during the process.

## 6 Operation instructions

This section gives detailed instructions on how to operate the motorized rotation stage. It includes the basic setup, 1 inch optics mounting, a minimal working example and an overview of the implemented commands. The prior installation as described in Section 5.4 is mandatory and presumed. First, read the entire section to avoid possible safety hazards.

### 6.1 Operation precautions

To prevent the stepper motor from overheating or burning out, the maximum current per phase of the TMC2209 must be reduced. While the stepper motor used is designed for a current of 0.67 amps per phase, the motor driver used can potentially provide up to 2 amps per phase. The factory setting is 0.9 amps per phase and should therefore be reduced before initial start-up. To do this, the potentiometer (the small screw) on the motor driver must be turned clockwise until the voltage between the screw and ground is approximately 0.85 V (1.2 V by default).

In addition, the free movement of the rotation stage must be checked before the initial start-up. Poor alignment of the gear wheel on the motor side, a belt tensioner that is too tight, or other blockages in the belt drive can cause the motor to stall. In this case, the motor permanently applies its maximum torque and heats up accordingly. One possible consequence of this is the potential softening of the plastic housing, whereby Green TEC PRO was selected as a filament as a precautionary measure to withstand the high temperatures. If required, the housing can also be printed from ABS as an alternative. Regardless of the stepper motor’s high operating temperature, its maximum ambient temperature rating of 50 °C combined with a maximum relative humidity of 85% defines the rotation stage’s operating conditions and should not be exceeded.

### 6.2 Remote control

**Fig. 4:**
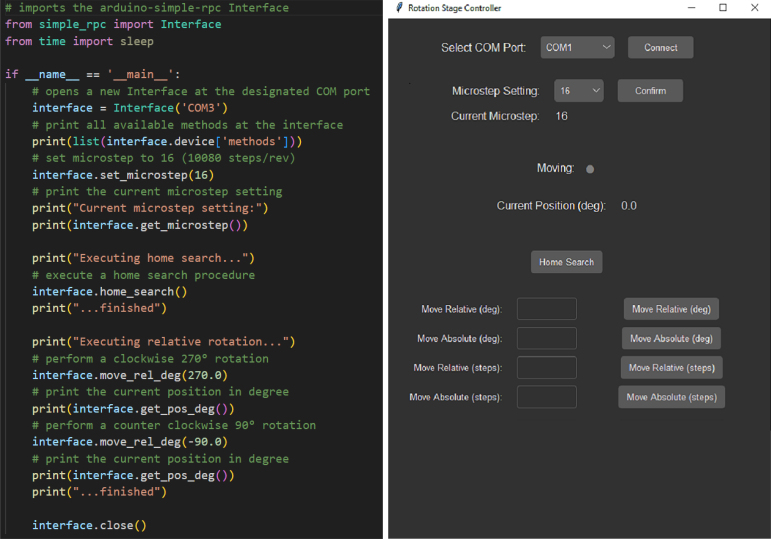
Left: Screenshot of the python minimal working example code, displaying the available methods at the interface on the console, as well as performing a home search and two relative movements (＋270° and −90°). Right: Screenshot of the GUI interface for manual control of the rotation stage. Users can select the appropriate COM Port from a dropdown menu and set the microstep setting as needed. The GUI provides access to basic movement functions, including a home search routine, and displays the current position in degrees. A movement indicator lights up during active movement.

First connect the rotation stage to your computer via the USB-C connector. Afterwards, plug in the 12 V DC power supply and make sure to comply with Section 6.1 to avoid any damage to the motor or rotation stage. To remotely control the rotation stage, install python and choose the distribution of your preference (i.e. VS Code). Ensure that the version used is not below 2.9.12, as this was used for testing purposes. The following steps have to be performed to set up the remote operation:

1. Install the simple-RPC (pip install arduino-simple-rpc) and the pyserial (pip install pyserial) library from PyPI.
2. Open the python script ESP32_MotionCTRL_serialPy.py shown in Fig. 4 (Left).
3. Make sure to set the correct COM port that connects to the microcontroller.
4. Execute the script. A comprehensive test routine is run. In addition, an overview of the available RPC calls is listed in the prompt line.

The rotation stage can now be controlled remotely using Python commands. The detailed command structure with corresponding input and output parameters is provided in Appendix C, or can alternatively be queried with a Python prompt. For day to day operation and optical alignment, a GUI (cf. Fig. 4 (Right)) can be used for manual control of the rotation stages rudimentary functions. These include the movement (relative/absolute movement in steps and degree), homing, and microstep settings of the stage. The GUI operates on the basis of pythons tkinter package and can be started by running the python script RotationstageGUI.py. The correct COM port has to be chosen in a dropdown menu listing all available COM ports, to connect to the rotationstage. Alternatively, to manually move the stage, the two buttons on the stages side panel can be used. The left button is designated for counterclockwise rotation, whereas the right button rotates the stage clockwise.

### 6.3 Optics mounting

The optics holder of the rotation stage is explicitly designed for housed 1 inch optics such as polarizers or wave retarder plates. To install an optic, it must be pressed into the holder. A special tool to facilitate insertion and removal can be found under ’Optic_Tool’ in the design files. It is specially designed to rest solely on the edge of the optics housing. The tolerances of the holder are designed in such a way that the housed optics are held in place as a press fit. The flexibility of the plastic used contributes to this.

## 7 Characterization

In this section, the performance of the designed motorized rotation stage is analyzed and demonstrated by means of spatial detection employing a line scan camera and simple geometry. The parameters characterized include the bidirectional repeatability (backlash), absolute positioning accuracy (precision), home positioning accuracy and single step resolution.

### 7.1 Optical characterization setup

**Fig. 5:**
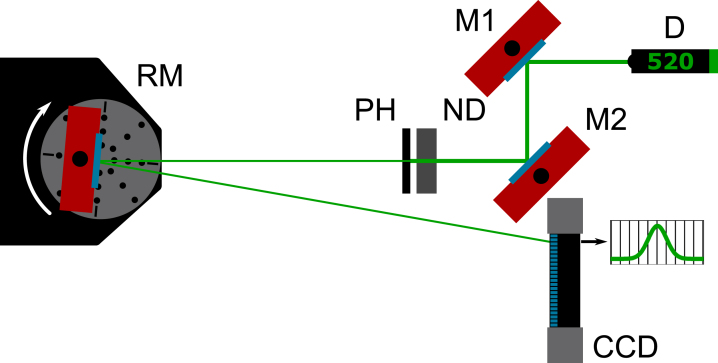
Setup for the characterization of the rotation stage’s performance. It consists of a diode laser (D), two adjusting mirrors (M 1,2), a neutral density filter (ND), a pinhole (PH), a mirror (RM) and a linear CCD array (CCD).

The experimental setup employed to characterize the rotation stage is shown in Fig. 5 and utilizes the rotation stage in the platform configuration. It consists of a laser source (‘Thorlabs, Inc.’, type PL201, 0.9 mW @ 520 nm), a neutral density filter (‘Thorlabs, Inc.’, type NE20 A, OD 2.0), a pinhole (‘Thorlabs, Inc.’, type P500K, d=500μm) and the rotation stage with a silver mirror mounted on the rotation axis (‘Thorlabs, Inc.’, type PF10-03-P01, protected silver). A line scan camera (‘EURECA Messtechnik GmbH’, type e9u-LSMD-TCD1304-STD, 3648 pixel @ Δd =8μm) is used to measure the horizontal position of the incident laser beam. By rotating the silver mirror, the reflected laser beam moves horizontally along the pixels of the line scan camera. A shift in the position of the sinc${}^{2}$ intensity distribution, due to diffraction of the light at the pinhole, can be measured. The absolute angular orientation of the rotation stage can be calculated using the geometric relationship between the horizontal and vertical distances between the axis of rotation of the mirror and the line scan camera.

### 7.2 Backlash characterization

**Fig. 6:**
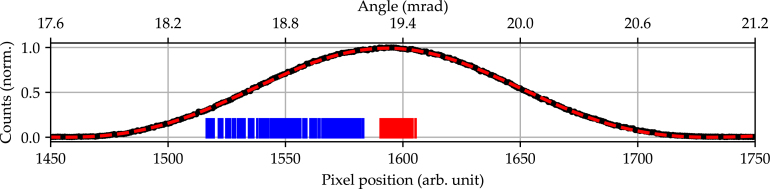
Backlash measurement performed over a total of 200 iterations. The incident laser is aligned with the center of the line scan camera, while for each iteration an alternating rotation of −360° and ＋360° is performed. Sinc${}^{2}$ modeled positions of the incident laser spot on the line scan camera.

The backlash between different parts of a mechanism, here the gears and belt, is defined as the clearance between those parts. This phenomenon is most noticeable at the beginning of a bidirectional rotation sequence, where a change in rotational direction is met with a small angular range in which no movement gets transmitted from the motor to the rotation axis. To characterize this backlash, the incident laser is aligned with the center of the line scan camera, while for each iteration an alternating rotation of −360° and ＋360° is performed. After each full rotation, the mean position of the laser beam is measured. The deviation between these two positions is determined in order to calculate the resulting clearance. This procedure is repeated for a total of 200 iterations in order to acquire statistically significant results. In Fig. 6) the backlash analysis, which was measured at a microstep setting of 64, is shown.

**Fig. 7:**
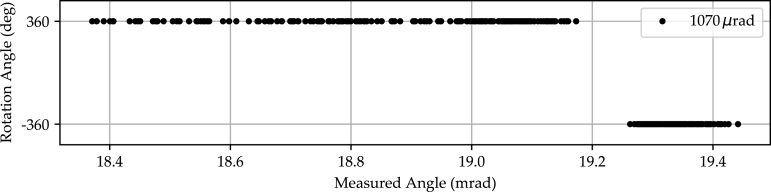
Geometrically derived angular position of the −360° and ＋360° rotation. The maximum angular distance of 1070 μrad corresponds to the backlash.

The graph shows an example of the intensity profile cross-section acquired with the line scan camera (black dots) and the corresponding sinc${}^{2}$ fit (black dashed line). For the remainder of the measurement data, the resulting mean pixel position is indicated by blue and red lines. Here, blue lines correspond to the ＋360° position and red lines to the −360° position. The graph in Fig. 7) additionally shows the statistical parameters of the mean and peak values of the two accumulation points. The backlash of 1070 μrad derived from this measurement is the maximum angular distance measured between the −360° and ＋360° positions. The average backlash per iteration is 445 μrad with a standard deviation of 197 μrad. For a conservative estimate of the stage’s backlash characteristic, the maximum backlash across all iterations is used as a reference.

### 7.3 Home positioning accuracy

**Fig. 8:**
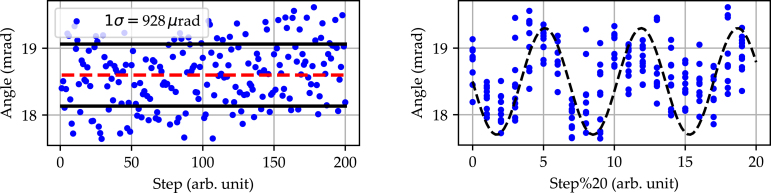
Home positioning accuracy performed over a total of 200 iterations. For each iteration a new home search procedure is performed, followed by a fixed, predetermined offset to align the incident laser with the center of the line scan camera. Left: Shown is the geometrical derived angular position for each iteration with the corresponding mean and standard deviation. Right: Shown is the geometrical derived angular position for each iteration modulo 20, revealing a sinusoidal pattern.

Similar to the backlash, the contact point at which the built-in home switch triggers, and thus the angular position, is subject to inaccuracy and must therefore be characterized. To determine the home positioning accuracy, the rotation stage performs a home search procedure followed by a fixed, predetermined offset to align the incident laser with the center of the line scan camera. This process is repeated for a total of 200 iterations to acquire statistical information on the trigger point. The overall homing accuracy is then defined as the 1 σ environment of the acquired distribution. Fig. 8 depicts the determination of the home positioning accuracy exemplarily for a microstep setting of 64.

The graph on the left shows the geometrical derived angular position for each iteration. Additionally, the mean with its corresponding 1 σ environment is indicated. The home positioning accuracy determined from this measurement is 928 μrad.

A closer look at the left graph reveals a pattern in the distribution of the measured points. If the ascending number of iterations is divided by modulo 20 and the points with identical iterations are plotted on top of each other, the graph on the right is derived. It shows a clearly pronounced sinusoidal relationship, which is repeated for every 20 full rotations. The reason for this is the shape tolerance of the pulleys used. With the selected transmission ratio of 20 to 63, both pulleys are only in the same position relative to each other after 20 full revolutions. In principle, it is possible to carry out an additional calibration based on this information, the home switch and the encoder, which reduces the home positioning accuracy to the amplitude noise of the sinusoidal function. However, as this calibration depends on the individual tolerances of the respective rotation stage and cannot be carried out in advance, it is not included in this work.

### 7.4 Precision measurement

**Fig. 9:**
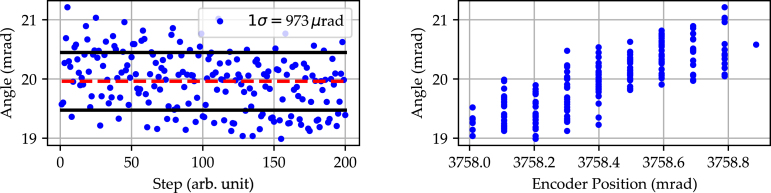
Precision determination performed over a total of 200 iterations. The incident laser is aligned with the center of the line scan camera, while for each iteration a relative 360° rotation is performed. Left: Shown is the geometrical derived angular position for each iteration with the corresponding mean and standard deviation. Right: Shown is the correlation between the encoder position and the geometrical derived angular position.

The ability to repeatedly move to a fixed position is an elementary characteristic of a rotation stage and is referred to as precision. To determine the rotation stage’s precision, the incident laser is aligned with the center of the line scan camera. For each iteration a full rotation is performed. After each full rotation, the angular position is determined geometrically. This process is repeated over 200 iterations. From this, the mean value and standard deviation of the measured positions on the line scan camera is determined to estimate the rotation stage’s precision. The respective analysis is shown in Fig. 9.

The graph on the left shows the geometrically derived angular position for each iteration. Additionally, the mean with its corresponding 1 σ environment is indicated. The precision determined from this measurement is 973 μrad. The graph on the right shows the correlation between the encoder position and the geometrical derived angular position. A decreasing linear relationship can be seen, which indicates that a spatial deviation on the line scan camera is only partially mapped by the encoder. The reason for this is the direct coupling of the encoder with the motor, which means that tolerances in the transmission between pulley and belt are not accounted for. It should be noted that this characterization also shows a modulation of the measured position every 20 revolutions.

### 7.5 Resolution measurement

**Fig. 10:**
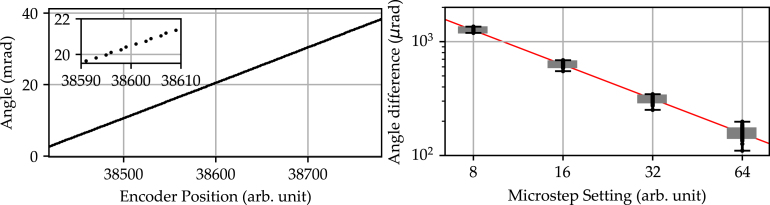
Resolution measurement performed over the entire active area of the line scan camera (≈ 3648 pixel over 29.1 mm). Left: Shown is the correlation between the encoder position and the geometrical derived angular position. The inset provides a detailed view over a range of 20 successive steps. Right: Shown is the angular difference between two successive steps (black dots). The minimum and maximum angular increment is indicated by the error bars, while the gray box highlights the 1σ environment. The measurement was performed for the full set of microstep settings (8-, 16-, 32- and 64-fold microstepping).

The rotation stage’s resolution is a measure for the minimum incremental motion of the stage that can be repeatedly set. For the characterization procedure, the stage is rotated in single steps, with the beam traversing the full width of the line scan camera. This incremental movement amounts to a total angular rotation of the stage by approximately 2°. The corresponding analysis is depicted in Fig. 10.

The left graph shows a causality between encoder position and real, measured angular position. In particular, each step can be clearly differentiated. Despite the clear differentiability of two successive steps, the inset shows that the incremental rotation remains, to a minor degree, susceptible to step-to-step variation. In addition to the inherent inaccuracies of the stage due to mechanical tolerances, this can be partly explained by the mismatch of the encoder steps and motor steps. As the sampling rate of the encoder is below the desired Nyquist limit (20480 encoder steps to 12800 motor steps), this leads to discrete jumps in the mapping of motor steps to encoder steps. A more detailed, statistical analysis is presented in the right graph. Here a full presentation of each step-to-step difference at the respective microstep setting (8-, 16-, 32- and 64-fold microstepping) is shown. The 1σ environment is indicated by a gray box, with the error bars defining the minimum and maximum step difference. The double logarithmic plot shows the expected linearized relationship between microstepping and incremental step resolution, as indicated by the red line. A detailed list of the mean increments and associated standard deviations resulting from this analysis is given in Table 3.

### 7.6 Microstep settings

The previously described characterization of the relevant properties of the rotational steps was carried out for the various microstep settings provided by the motor controller. In total, four different settings can be applied, including 8-, 16-, 32- and 64-fold microstepping. Combined with the gear ratio of the systems, the theoretically feasible minimum rotation increment amounts to the 1/14, 1/28, 1/56, and 1/112 part of a degree. The respective performance of each setting, measured in accordance to the previously introduced methodologies, is listed in Table 3.

**Table 3:** Listed are the experimentally determined Resolution, Precision, Backlash and Homing for the 8-, 16-, 32- and 64-fold microstepping.

Microstep setting	Resolution	Precision	Backlash	Homing
8	1268±45μrad	977μrad	686μrad	1154μrad
16	633±26μrad	983μrad	584μrad	984μrad
32	314±16μrad	1017μrad	1175μrad	920μrad
64	158±13μrad	973μrad	1070μrad	928μrad

### 7.7 Key specifications

In Table 4, the key specifications of the rotation stage are highlighted. For comparison, the corresponding specifications of similar open-source and commercial devices are shown.

**Table 4:** Highlighted are the key specifications of the rotation stage in comparison to similar open-source and commercial alternatives.

**Manufacturer**	This work	Appl. Optics [22]	Thorlabs [9]

**Version/Model**	64 step	256 step	K10CR1
**Optic Mount**	1 inch	1 inch	1 inch
**Dimensions**${}^{1}$	87 × 88 × 74 mm${}^{3}$	85 × 40.5 × 102.5 mm${}^{3}$	21.5 × 107×66 mm${}^{3}$
**Motor type**	Stepper motor	Stepper motor	Stepper motor
**Gearing**	3.15:1	2:1	120:1
**Velocity**	0.62rad/s	7.85rad/s	0.175rad/s
**Torque**	0.3N m	0.7N m	0.14N m
**Resolution**	158±13μrad	70±30μrad	524μrad
**Precision**	973μrad	600μrad	±60μrad
**Backlash**	1070μrad	700μrad	±200μrad
**Homing**	928μrad	−μrad	±100μrad
**Integrated Controller**	yes	no	yes
**Unit price**	100Euro	220–270Dollar	1400 Euro (excl. VAT)

			

## 8 Selected applications

In this section, selected applications of the designed motorized rotation stage are demonstrated by means of laser intensity regulation and the writing and readout of an elementary holographic grating. Both applications are typical examples in the field of photonics, which feature a broad range of rotational precision requirements. At the same time, they cover both measurement configurations of the motorized rotation stage.

### 8.1 Laser intensity regulation

**Fig. 11:**
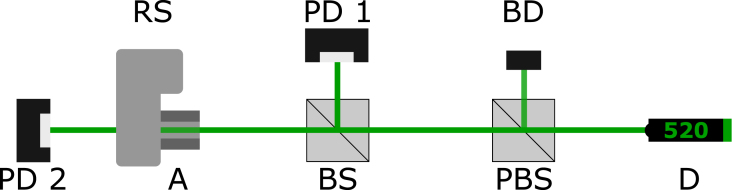
Setup for the characterization of the rotation stage’s performance. It consists of a diode laser (D), a polarizing beamsplitter (PBS), a beam dump (BD), a 50:50 beamsplitter (BS) and an analyzer Left:, rotation stage (RS) and two photodiodes (PD 1,2).

Laser intensity regulation and polarization adjustment are both common and closely related applications that are present in almost all optical experiments. The most common setups use a combination of rotating polarizers and/or wave retarder plates, although one of the optical components may be sufficient depending on the specific application. For this reason, cost-effective automation of these two components is of particular interest if they often have to be varied manually. The setup employed in this work aims to regulate the intensity of the given laser source and is shown in Fig. 11.

This setup consists of a laser source (‘Thorlabs, Inc.’, type PL201, 0.9 mW @ 520 nm), a beamsplitter (‘Thorlabs, Inc.’, type BSW10R, 50:50), a polarizing beamsplitter (‘Thorlabs, Inc.’, type PBS121) and the rotation stage with an integrated analyzer (‘B.Halle Nachfl. GmbH’, type PGT 2.12, Glan–Thompson-Prism). Two photodiodes (‘Thorlabs, Inc.’, type S120VC) are used to measure the reference power reflected by the beamsplitter as well as the power transmitted through the analyzer. By rotating the analyzer’s optical axis with respect to the polarizing beamsplitter’s optical axis, the power transmission through the analyzer can be varied according to Malus law:

$$P=P_{0}\;\cos^{2}\theta$$

Here P is the power transmitted through the analyzer, $P_{0}$ is the initial power after transmission through the polarizer and θ is the angle between the polarizer’s and the analyzer’s optical axis. In this definition θ=0 corresponds to the extinctions minimum, i.e., a maximum in transmission.

**Fig. 12:**
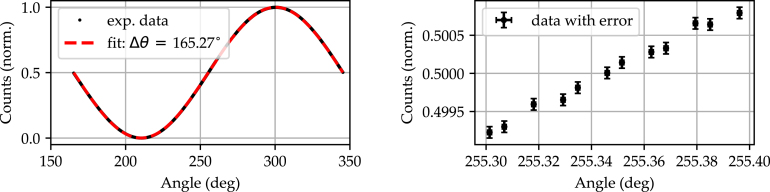
Left: Measurement of a 180° rotation of the analyzer at a fixed polarizer position. Right: Close up analysis of the first infliction point in the vicinity of the 255.35° position of the analyzer. Error bars indicate the 2σ range of laser power fluctuation determined by the stability measurement (Appendix A).

To showcase the system’s performance, a full cycle rotation is measured at full resolution, with a single rotation increment of 1/112°. The measured signal transmission as a function of the polarizer’s angle of rotation is depicted in Fig. 12. In addition, a close up view of the transmission in the vicinity of the inflection point at the 254° position is shown.

While the individual steps cannot be differentiated in the selected window of Fig. 12 Left, the fit function used clearly shows the idealized curve from 165° to 345°. This kind of measurement also allows for the positional calibration of the polarizer used. In this case, an offset of 165.27° relative to the aspired position was determined. In combination with the built-in home switch and this offset, it is possible to move repeatedly to a defined position and thus to adjust the transmitted intensity. The selected window in Fig. 12 Right shows that the rotation of the polarizer and the associated intensity modulation can be resolved with a resolution of 1/112°.

### 8.2 Reconstruction of holographically recorded elementary volume phase gratings

The reconstruction of holographically recorded volume phase gratings provides an ideal tool for the validation of the functionality of the rotation stage in its platform configuration. The tool makes use of the high angular sensitivity of the Bragg condition in the optical domain for diffraction gratings with large thickness. We here use a two-beam interferometer that allows both, the recording and reconstruction of elementary holographic gratings in one and the same setup. It consists of two mutually coherent laser beams, the so-called reference and signal beams, which are superimposed within a photosensitive material to generate a spatially modulated sinusoidal intensity pattern. Bright regions of the fringe pattern are transferred into a change of the absorption coefficient and/or refractive index in photochromic and/or photorefractive materials, respectively, while the material in the dark areas remains unchanged. A spatially modulated absorption and index pattern results, which is referred to as elementary holographic absorption (or lossy) and phase grating, respectively. They are labeled as thin or thick depending on whether higher diffraction orders are generated or suppressed.

Thick (or volume) gratings recorded in this way make it possible to reconstruct the signal beam by illumination of the grating with the reference beam, only, and vice versa. It is because the Bragg condition of the grating is automatically fulfilled by both of the recording beams. Alternatively, a laser beam detuned in its wavelength with respect to the recording wavelength can be used, as well, provided that the Bragg condition is maintained by appropriate angular adjustment. This makes it possible to reconstruct with wavelengths at which the recording material is insensitive to light exposure, i.e. the hologram is not destroyed during reconstruction.

Using a motorized rotation stage, the angular sensitivity of the recorded grating, i.e. the sharpness of the Bragg condition, can be inspected by angular detuning. We here note that the sharpness increases with increasing grating thickness so that it is possible to estimate the depth of the recorded hologram within the photosensitive material. For this purpose, the break-down of the intensity of the reconstructed signal beam is measured as a function of an angular mismatch ±Δθ with respect to the Bragg incidence at angle $\theta_{\mathrm{Bragg}}$, i.e. a so-called Rocking curve results. Taking into account that the angular sensitivity is much below 1/10 degrees for commonly used photosensitive materials, an angular sensitivity of the rotation stage of 1/100 degrees is inevitably required to determine the Rocking curve with sufficient precision. In addition, high-contrast holographic recording requires a high degree of mechanical stability of the rotation stage throughout the recording process with interferometric precision.

**Fig. 13:**
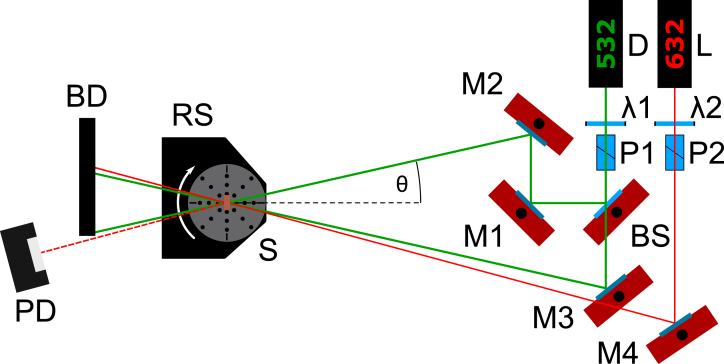
Setup of the two-beam interferometer for the validation of the functionality and performance of the rotation stage. It consists of a Nd:YAG and He–Ne laser (D,L), a combination of half-wave retarder plate (λ1,2) and Glan–Thompson polarizers (P1,2), four adjusting mirrors (M1.M4), a 50:50 beamsplitter (BS), a beam dump (BD), a motorized rotation stage (RS), a photodiode (PD) and a Fe-doped Lithium Niobate sample (S).

The two-beam interferometer setup employed for the recording of the holographic grating as well as for the determination of the Rocking curve with the motorized rotation stage is schematically depicted in Fig. 13. A photorefractive single crystal of Fe-doped Lithium Niobate (LiNbO${}_{3}$, LN) with pronounced photosensitivity in the blue–green spectral range served as holographic recording material for our experiments [27]. This setup consists of two lasers, one for recording at a wavelength of λ=532  nm (‘Pegasus Optik’, DPGL-2150, 150 mW) and one for reconstruction at λ=632.8  nm (‘Melles Griot’, 05-LHP-991, Helium–Neon Laser, 10 mW). Intensity and light polarization are adjusted individually by a combination of half-wave retarder plates and Glan–Thompson polarizers (’B.Halle Nachfl. GmbH’, type PGT 2.12, Glan–Thompson-Prism). An intensity ratio of 100:1 was chosen for the recording and reconstruction beams. Reference and signal beam were generated via a 50:50 beamsplitter (‘Edmund Optics Inc.’, type 43-736). The photorefractive LN crystal (thickness ≈0.2mm, Fe concentration ≈0.1%  mol) was mounted onto the rotation stage with a 3D printed sample holder. A photodiode (‘Thorlabs, Inc.’, type S120VC) is used to measure the intensity of the diffracted beam of first order, i.e. the intensity of the reconstructed signal beam. An (externally measured) Bragg angle of ≈5 degrees is chosen for hologram recording and the angle of the reconstruction beam is adjusted to fulfill the Bragg condition.

**Fig. 14:**
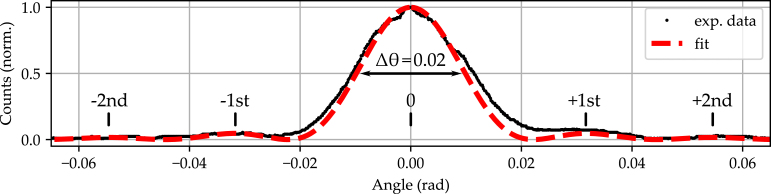
Rocking curve acquired in LiNbO${}_{3}$:Fe (thickness ≈0.2mm, Fe concentration ≈0.1%) for an angular detuned reconstruction of a holographic grating within an angular detuning range of ±0.06 rad and at an incremental motion of 156 μrad. A sinc${}^{2}$ function (red dashed line) was fitted to the experimental data (black dotted line) in accordance with the coupled-wave theory for elementary holographic volume phase gratings diffraction.

**Fig. 16:**
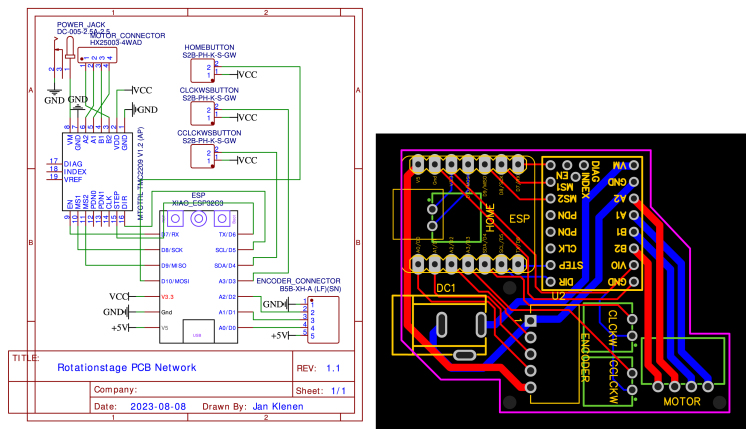
Left: Wiring diagram for the rotation stage. Right: corresponding PCB layout for the rotation stage.

The holographic grating was recorded for a duration of approximately 500 s with an intensity of ≈50  mW/cm${}^{2}$ per beam (polarization parallel to the polar c-axis of the crystal). At the end of the recording process, the 532 nm laser light was blocked and the signal beam was reconstructed with the 632 nm laser light. Then, the Rocking curve was determined by performing an angular scan over an angular range of ≈±3.4 degrees (≈±0.06  rad) with respect to the Bragg angle. The rotation speed was kept constant with incremental steps of 156 μrad, which corresponds to a resolution of 64-fold microstepping. The determined experimental data for the intensity as a function of angular detuning is shown in Fig. 14.

The experimentally determined data points are depicted in black and show a distinct maximum at Bragg incidence (≡Δθ=0 degrees) with a full width at half maximum of FWHM=0.02 rad. Furthermore, the ± first and ± second side maxima of the angular distribution can be clearly identified. A sinc${}^{2}$ function is fitted to the data set (red dotted line) which shows a very good agreement between theory and experiment. Minor deviations from the theoretical function, such as the appearance of slight shoulders in the reconstruction peak, may be attributed to the presence of a non-sinusoidal index grating as a result of a slight overexposure in the recording process. The possibility to uncover these aspects in addition to the determination of the width of the Rocking curve is regarded as successful proof for the functionality of the motorized rotation stage in this application example. We finally note that it is possible to inspect gratings with much higher angular sensitivity, as well, taking the maximum available angular resolution of 156 μrad into account.

## CRediT authorship contribution statement

**Yannic Toschke:** Writing – review & editing, Writing – original draft, Visualization, Validation, Hardware, Software, Methodology, Investigation, Formal analysis, Data curation, Conceptualization. **Jan Klenen:** Writing – review & editing, Writing – original draft, Visualization, Validation, Hardware, Software, Methodology, Investigation, Formal analysis, Data curation, Conceptualization. **Mirco Imlau:** Writing – review & editing, Supervision, Resources, Project administration, Funding acquisition.

## Declaration of competing interest

The authors declare that they have no known competing financial interests or personal relationships that could have appeared to influence the work reported in this paper.

## Figures and Tables

**Fig. 15:**
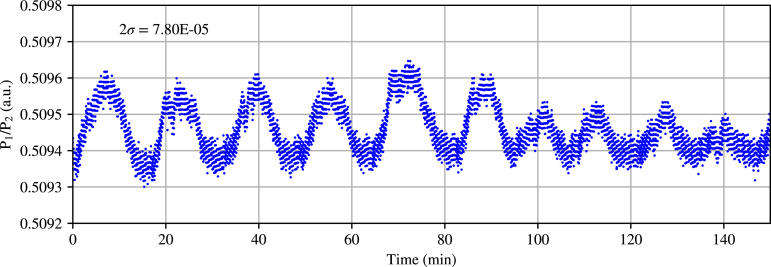
Long term stability measurement of the corrected laser power $P_{\mathrm{D2}}$/$P_{\mathrm{D1}}$ for 100-fold averaging of the detectors, measured over a period of 150 min. The polarizer and analyzer are aligned in correspondence to the later used measurement configurations, i.e. the analyzer is tilted by 45° relative to the polarizer. The floating 2σ range of the standard deviation was determined and added to the graph.

**Figure:**
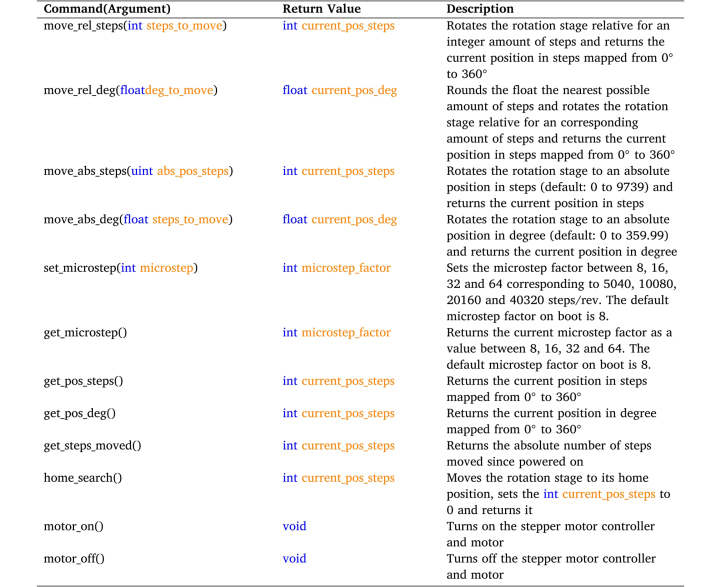


## Appendix A Long-term stability of the measurement setup

As the determination of the rotation stage’s usability for intensity regulation requires the detection of minuscule changes in the transmission of the laser power at the detector $P_{D1}$, the signal-to-noise ratio of the setup has to be characterized comprehensively, especially with regard to long-term stability. There exists a broad range of factors that have significant influence on the overall noise of the detection, the most important ones are listed below:

• Temperature variations of the laser diode
• Instabilities of the power supply
• Ambient influences (air temperature, humidity)
• Mechanical vibrations
• Beam pointing fluctuations
• Detector noise (thermal noise, shot noise, etc.)

The former two factors, temperature variations of the laser diode and instability of the power supply, are corrected by normalizing the transmission signal of $P_{D1}$ to the reference signal of $P_{D2}$. To further stabilize the system, the ambient temperature is regulated, and the setup is placed on a pneumatically damped optical table. Despite these measures, the measurement remains inherently prone to noise, as for example the detector noise, beam pointing fluctuations, as well as remaining vibrations and temperature fluctuations that cannot be fully suppressed influence both detection paths independently of one another and thus limit the overall signal-to-noise ratio of the system. Characterization of the long-term stability of the measurement is therefore required in order to evaluate if the signal-to-noise ratio is sufficient to resolve a single step of the rotation stage. The measurement is performed in the vicinity of the inflection point of the transmission curve, as this is where the power change per step is the greatest and consequently the detection sensitivity is the highest. This condition is fulfilled at a polarizer position of 45°. At this point, the change in transmission for a single step (1/122°) equates to:

(1) $$\frac{\partial P}{\partial \theta }=-2P_{0}\cdot \cos\left(\theta \right)\sin\left(\theta \right)=-P_{0}\cdot \sin\left(\theta \right)$$

(2) $$\frac{\Delta P_{Step}}{P_{0}}=\sin\left(\theta +1/14{}^{\circ}\right)-\sin\left(\theta \right)$$

(3) $$\frac{\Delta P{\left(\theta =45{}^{\circ}\right)}_{Step}}{P_{0}}\approx 1,1\cdot 10^{-4}$$

Hence, a relative power stability $P/P_{0}$ on the order of 10^−4^ is required to resolve a single step of the rotation stage. For verification purposes, the power stability of the system at a fixed polarizer position of 45° is measured over a time span of 150 min. To improve the signal-to-noise ratio, 100-fold averaging is performed per acquired data point. The evaluation of the results is shown in Fig. 15.

The range of variation of the laser power measured at the 2 sigma environment is $3\cdot 10^{-4}$. This value exceeds the power stability required to resolve a step. However, a full rotation of the rotation stage only requires a few seconds. Hence, the moving power stability on a lower timescale of a minute is of relevance for the evaluation. This amounts to $7.8\cdot 10^{-5}$, which is within the required tolerance range to resolve the smallest microstep increment.

## Appendix B Electrical wiring diagram and PCB layout

See Fig. 16.
